# Gender differences in clinical presentation and therapeutic response in schizophrenia

**DOI:** 10.1192/j.eurpsy.2025.2255

**Published:** 2025-08-26

**Authors:** S. Ben Aissa, K. Razki, C. Najar, A. Larnaout, W. Melki

**Affiliations:** 1 Psychiatry, Razi hospital, Manouba, Tunisia

## Abstract

**Introduction:**

Schizophrenia is a multifaceted psychiatric disorder characterized by disturbances in thinking, emotions, and behavior. It exhibits a diverse spectrum of clinical presentations influenced by various factors, among which gender plays a significant role.

**Objectives:**

To determine the differences in clinical profile and therapeutic response among schizophrenia patients according to gender.

**Methods:**

We conducted a descriptive, cross-sectional, comparative study over a three-month period from January to March 2024, among patients in the post-care service of Psychiatry Department D at Razi Hospital diagnosed with schizophrenia based on DSM-5 criteria. Sociodemographic and clinical data were initially collected from patients’ medical records and then verified and supplemented during direct interviews using a pre-established information sheet. The Positive and Negative Syndrome Scale (PANSS) was used to assess symptom severity in participants.

**Results:**

We enrolled 80 participants, of whom 50 were male (62.5%) and 30 were female (37.5%). The mean age of participants was 42.5 years. Men had higher mean scores on the positive symptoms subscale (75.6±12.05) and negative symptoms subscale (64.8±8.69), while women had higher mean scores on the general symptoms subscale (69.3±15.86). Male patients exhibited a higher prevalence of auditory hallucinations (80%) compared to women (55%), with a statistically significant difference (χ² = 4.32, p < 10-3). Similarly, a higher prevalence of delusional ideas (72%) was observed in men compared to women (45%), also statistically significant (χ² = 5.87, p = 0.02). Regarding therapeutic response, men showed a statistically more significant improvement in positive symptoms like hallucinations and delusional ideas, with an average reduction of 15 points on the PANSS scale compared to 10 points in women (p = 0.02). Conversely, women demonstrated a more favorable response to negative symptoms such as social withdrawal and apathy, with an average reduction of 12 points on the PANSS negative symptoms subscale compared to 8 points in men (p = 0.04).

**Conclusions:**

The observed differences in clinical profiles and therapeutic responses between male and female patients underscore the necessity for tailored treatment approaches aimed at optimizing outcomes and enhancing patient care.

**Disclosure of Interest:**

None Declared

